# Elements of successful patient involvement in clinical cancer trials: a review of the literature

**DOI:** 10.1016/j.esmoop.2024.102947

**Published:** 2024-03-15

**Authors:** I. Shakhnenko, O. Husson, D. Chuter, W. van der Graaf

**Affiliations:** 1 European Organisation for Research and Treatment of Cancer (EORTC Headquarters), Brussels, Belgium; 2 Department of Medical Oncology, Netherlands Cancer Institute, Antoni van Leeuwenhoek, Amsterdam; 3 Department of Surgical Oncology, Erasmus MC Cancer Institute, Erasmus University Medical Centre, Rotterdam, The Netherlands; 4 EORTC, Patient Panel, Brussels, Belgium; 5 Digestive Cancers Europe (DiCE), UK; 6 Department of Medical Oncology, Erasmus Medical Centre Cancer Institute, Erasmus University Medical Centre, Rotterdam, The Netherlands

**Keywords:** patient involvement, clinical trials in cancer, patient involvement in cancer research, successful patient involvement practice, meaningful patient involvement

## Abstract

Patient involvement in clinical cancer research has gained much ground in the past few years and studies demonstrated positive outcomes of such involvement. Yet, they also indicated a lack of evidence around best methods and practices to achieve successful patient involvement. The aim of this literature review was to provide a synthesis of elements contributing to successful and meaningful ways of involving patients in oncology trials across different stages of research. This synthesis can offer practical support to researchers in their patient involvement journey. A PubMed literature search for original articles published between 2012 and early 2023 was carried out. In total, 3132 articles were identified, among which 152 were fully assessed for eligibility. Thirty-three articles met the predefined inclusion criteria and were subjected to a quality checklist. Patient involvement occurred most often in the development stage of cancer trials (85%) and was continuous and integrated throughout the entire lifecycle of research (67%). In total, 58 elements of successful patient involvement were identified, such as clearly defined roles and responsibilities of patient partners, input of multiple patients to ensure diversity, and regular touchpoints in the project. All these elements can be applied in future studies from the planning stage to the dissemination of study results. This review provides a set of practical recommendations that can be used by the cancer research community when planning to involve or already involving patients in their clinical trial activities.

## Highlights

• This review provides a synthesis of elements contributing to meaningful ways of involving patients in oncology trials.
• Collecting patients’ input from the onset of the study helps achieve successful and purposeful collaborations.
• Understanding patients’ personal experiences and motivations to contribute to the study helps to select suitable candidates.
• Patients’ roles and responsibilities should be clearly defined before their involvement.
• Training and supporting information materials for patients are essential for successful patient involvement.

## Introduction

Over the past two decades, patient involvement has gradually become a vital component of cancer research and has proven its multiple benefits. Perspectives of patients and caregivers on various research activities have helped define research priorities, eliminated studies irrelevant to patients, improved the overall quality of research, raised awareness around patients’ needs and rights, and, as a result, shaped a more patient-centred research environment where the role of the cancer patient shifted from merely a research participant to an active and involved research partner.^1^, ^2^, ^3^, ^4^, ^5^

However, despite its obvious value for the entire cancer research community represented by professionals, as well as patients as end-users, challenges and uncertainties around involvement are still present. Oftentimes, these challenges are commonly recognised and may vary from the difficulty of finding patients and/or caregivers willing to get involved in research to the inability to integrate their feedback and objectively evaluate its impact on the trial.^2^^,^^6^, ^7^, ^8^, ^9^ Moreover, the physical condition of a patient partner or their close ones may change rapidly, which may lead to their unexpected (timely) unavailability and inability to contribute to the project.

Furthermore, not only is the entire clinical trial lifecycle complex and highly regulated, but also the process of involving patients means a time-consuming, emotional, and cognitive effort that requires patience and dedication from both researchers and patient partners, and the proper planning of resources from the very beginning of the project.^10^^,^^11^ Altogether, this may create a perception of patient involvement being rather challenging and, consequently, may decrease willingness among researchers to consult patients’ opinions when starting and conducting clinical trials.

The previously conducted studies aimed at patient involvement in cancer clinical research have already highlighted the need to involve patients and demonstrated the positive outcomes of such involvement.^3^^,^^9^^,^^12^, ^13^, ^14^, ^15^ Yet, they also have indicated the lack of research done on best methods and practices helping to achieve successful patient involvement. To address these limitations and explore potential solutions, there is a need to better understand differences in patient involvement practices applied at each clinical trial stage, from the study design to the dissemination of study results.

With this literature review, we aim to provide an extensive overview of patient involvement activities taking place at each stage of clinical trials. The overarching research question to be answered is: ‘What elements can contribute to successful and meaningful patient involvement practices?’ This review identifies several elements that can offer practical support to researchers on patient involvement in cancer clinical trials.

## Materials and methods

### Search strategy

For the literature review, we have selected papers that were published between January 2012 and March 2023 for the reason that patient involvement has only started to be frequently integrated into oncological trials in the past decade. To identify relevant papers, a computerised search of the literature was carried out with the PubMed search engine in January-March 2023.

The first search string was composed of combining terms related to ‘clinical trials’, ‘cancer’, ‘patient involvement’, and its alternatives, such as ‘patient engagement’ and ‘patient participation’.

The second search contained the key words ‘patient involvement in clinical trials in cancer’. Additionally, we created a third search string that contained the key terms mentioned above combined with ‘successful’ and ‘meaningful’. This was intended to better understand the perception of success and meaningfulness in the context of patient involvement in oncology.

### Inclusion and exclusion criteria

To be eligible for inclusion, articles needed to either present patient involvement experience in the whole lifecycle of clinical trials in cancer, whether one particular trial or a group of them, or at a certain stage of the trial—setting of research priorities, design, execution, or dissemination of study results.^16^ We are aware that patients are also involved in different regulatory bodies such as the European Medicines Agency or ethical committees. However, we only included patient involvement practices applied by the organisations conducting clinical trials, whether academic or industry-led.

Our search excluded articles on patient involvement in non-cancer studies and paediatric oncology. The latter decision is based on the fact that patient involvement in paediatric studies significantly differs from patient involvement in cancer studies for adults and is worth investigation in a separate review. Studies that seek to explore the views of patients and patient advocates on transforming cancer health care systems and policy were excluded from our review since they cover a wide range of activities. Lastly, we did not consider papers describing the set-up of projects of involving patients, i.e. projects that cannot demonstrate yet any evidence of patient involvement because they are currently under development.

As ‘patients’ is not the only category of individuals who are being involved in cancer research, we will be using the terms ‘patient partners’, ‘patients’, ‘caregivers’, ‘patient representatives’, and ‘patient advocates’ interchangeably in this review.

### Data extraction

In total, 3132 articles were identified through our search on PubMed. The first search yielded 227 articles, the second search 2865, and the last one 40 (Supplementary Table S1, available at https://doi.org/10.1016/j.esmoop.2024.102947).

**Figure 1:**
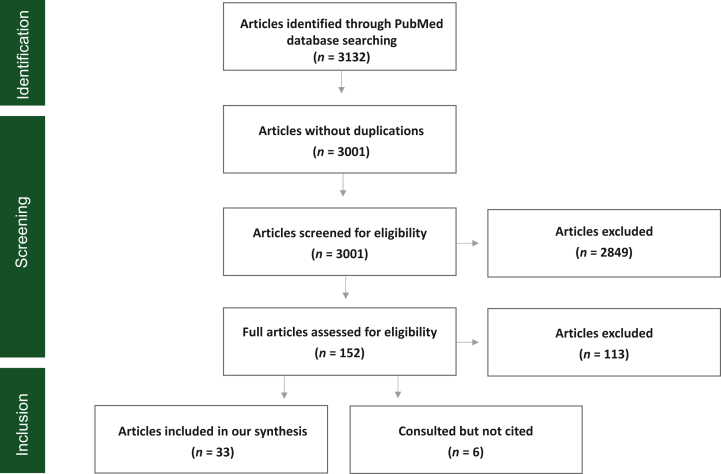
**Quality assessment.** We identified 3132 articles, of which 33 were found to be eligible and included in the synthesis.

After eliminating duplications and screening 3001 articles for eligibility, the search resulted in 152 articles that were read in full. Thirty-three articles fulfilled the eligibility criteria and formed the basis of our synthesis. In addition, six papers contributed to a better understanding of patient involvement in overall cancer research activities and were also used in this study (Figure 1).

For the analysis, we focused mainly on the characteristics of patient involvement at each trial stage and the elements that were deemed to contribute to the successful and meaningful practice of involving patients. Oftentimes, authors used words such as ‘principles’, ‘practices’, ‘approaches’, ‘mechanisms’, ‘models’, ‘strategies’, ‘frameworks’, and ‘recommendations’. Even though these words convey different meanings, all of them are being used to indicate a common attempt to illustrate examples of the successful application of patient involvement. For this review, and for the sake of consistency, we have chosen to use the term ‘element’ which refers to a rather smaller component of a broader concept.

## Results

Here we will first provide an overview of the studies that were included in our synthesis and then we will explain what patient involvement activities are typical for each stage and what elements can help improve patient involvement practice in oncological trials.

### Overview of studies

The findings revealed that the majority of articles (28 out of 33) shed light on patient involvement in the development stage when protocols and documents addressed to the patients are being drafted and when fundraising takes place. Sixty-seven percent of studies (22 out of 33) covered the application of patient involvement in the entire lifecycle of the trials. Less well investigated is patient involvement in the pre-development stage and in the final stage—dissemination of trial results, including publication and communication to trial participants (15 out of 33) (Supplementary Table S2, available at https://doi.org/10.1016/j.esmoop.2024.102947).

### Characteristics of patient involvement at each stage of cancer trials

#### Stage 1: pre-development

Fifteen out of 33 articles (45%) attempted to explore patient involvement in defining research priorities or so-called early involvement. A lesser number of articles addressing patient involvement in the early stages may result from a traditional approach to cancer research whereby investigators are the ones who define research priorities and formulate research questions, while patients associate themselves with study participants rather than co-creators of research. The inability to integrate the patient voice at this stage leads to overlooked research priorities and outcomes relevant to patients.^3^^,^^17^

Establishing partnerships between researchers and patients plays a significant role in developing, refining, and prioritising a research question or strategy.^18^ Patient voices not only help to prioritise the most important outcomes in the research but also help to choose the most appropriate definitions of those outcomes.^12^ For example, Batten et al. successfully demonstrated that patient perspectives included from the onset of a breast cancer trial provided reassurance about the acceptability of taking multiple biopsies. As hospitals and research teams tend to be reluctant to offer clinical trials requiring several tissue biopsies to recently diagnosed breast cancer patients, involvement of patient advocates was crucial. It helped clinicians to realise that their hesitancies to approach patients for trials involving multiple biopsies are often groundless and that there is a need to be transparent with potential trial participants about the rationale behind the tissue collection and the associated risks.^19^

#### Stage 2: study development

This stage mainly focuses on designing the study protocol, patient-facing documents, as well as raising research funds. Clearly, this is the most common stage of clinical trials where patients get involved in the following potential areas for collaboration: patient information sheet and informed consent, risks and benefits of the treatment, and recruitment strategy (28 papers out of 33).

While the complexity of the informed consent process can be a barrier to trial participation, one of the positive outcomes of patient involvement was the development of effective information materials that addressed the concerns of patients.^20^ Input from patient advocates on the development of patient information sheets and other patient-centred communication helps to ensure that the information is conveyed in an accessible and comprehensible format.^19^^,^^21^^,^^22^ They also brought ‘consent fatigue’ to researchers’ attention by highlighting the inconvenience of signing multiple forms by trial participants, insisted on simplifying the text, and co-developed a visual informed consent and non-technical summary of the consent document.^20^^,^^23^

The overall wording in patient-facing documents, including questionnaires, can be improved by patient partners’ feedback. For example, the question ‘Have you had radiation?’ may be understood not as a type of cancer treatment but as previously conducted tomography scans and thus lead to inaccurate responses.^17^ Also, the term ‘clinical trial’ has a rather negative connotation and may be associated with ‘randomised’, and therefore negatively impact enrolment in the study. Patient partners can also help research teams identify wording that patients may find confusing or unclear.^24^

Although recruitment to the trial starts later, involving patients at the stage of trial development is essential. Several studies illustrated that the early input of patients helped identify potential barriers and enablers to recruitment and retention linked to trial design or information provision.^8^^,^^20^^,^^25^, ^26^, ^27^, ^28^, ^29^ Early revision of trial design by patients can lead not only to an improved recruitment strategy but also to a successful application for research funding.^18^

#### Stage 3: execution

Sixty-seven percent of the included studies shed light on patient involvement activities taking place at the execution stage of clinical trials. The focus of this stage lies primarily on the recruitment and retention of trial participants, as well as data monitoring.

The study by Zaharoff et al. showed that collecting post-trial feedback from the participants can achieve better acceptance by patients and oncologists and benefit recruitment.^3^ The ability to identify issues experienced by patients participating in a trial, such as hospital location, fear of adverse events, lack of appreciation from clinical trial staff, and comprehensibility of risks and benefits, can help researchers improve patient experience during future trials.^30^

When it comes to trial recruitment of underrepresented minorities, there is a need for culturally appropriate strategies. The findings by Fouad et al. were crucial as they highlighted the benefits of support provided by patient partners to potential trial participants. Such support services included assistance with transportation and lodging, appointment reminders, and linking the patients with social and community services and resources. Beyond practical aspects, patient partners also offered social and emotional support, encouraged patients to share their concerns with their physicians, and helped navigate trial-related discussions. As a result of a successful integration of patient involvement, the study doubled the clinical trial completion rate of the trial participants.^31^

Even though patient involvement is often perceived as a promise to increase patient recruitment in oncological trials, success of trial recruitment is multifactorial: it also depends on patients’ awareness of and perceptions of clinical research, and the role of health care providers in delivering information about clinical trials to patients.^32^^,^^33^ The studies that we have included in this literature review demonstrated numerous benefits of patients’ input on trial recruitment; however, the relationship between the two seems to be rather indirect and requires an evidence-based approach.

#### Stage 4: dissemination of study results

Less than half of the included articles (45%) explored patient involvement in the final stage of clinical trials.

Involving patients in this stage makes the reporting of study results more meaningful and understandable for the research participants and the community.^2^^,^^18^^,^^20^ Patient partners can not only help with the dissemination of study results on websites and social media but also with the analysis and interpretation of the results.^34^ As an acknowledgement of patients’ contribution to the study, they may be included as co-authors of the publications.

The study by South et al. is worth noting as it considers co-publication with patients not only as an act of acknowledgement of their involvement but also as an opportunity for patients to share their perspectives on the side-effects of the investigated treatment. Such practice can help to place the study results in the context of real-life patient experience.^25^

### Elements of successful patient involvement

We propose to apply the elements that contribute to successful patient involvement practices at three different timepoints: during the creation of an environment for involving patients, in the process of and after their involvement. We also provide a list of the elements that are relevant to the entire patient involvement experience (Supplementary Table S3, available at https://doi.org/10.1016/j.esmoop.2024.102947). Fifty-eight elements of successful patient involvement practice were identified and can be utilised at any stage of the clinical trial.

This synthesis provides a wide range of recommendations for those who plan to or already involve patients in oncology trial activities. Some of these recommendations were highlighted by numerous studies and can be ranked, therefore, as the following ‘top-10’ elements:

1. Clearly defined roles and responsibilities of patient partners and expectations towards them^2^^,^^6^^,^^8^^,^^10^^,^^12^^,^^14^^,^^17^^,^^23^^,^^28^^,^^29^^,^^34^
2. Input of multiple patients to ensure diversity^1^^,^^2^^,^^6^^,^^7^^,^^9^^,^^14^^,^^15^^,^^17^^,^^22^^,^^25^^,^^35^
3. Inclusion of patient involvement in the budget^2^^,^^8^^,^^10^^,^^14^^,^^15^^,^^23^^,^^25^^,^^28^^,^^31^
4. Supporting tools and models for researchers^2^^,^^8^^,^^10^^,^^12^^,^^17^^,^^18^^,^^22^^,^^23^^,^^25^^,^^28^^,^^29^^,^^31^^,^^34^^,^^36^^,^^37^
5. Training and/or supporting information materials for patients^3^^,^^6^, ^7^, ^8^^,^^10^^,^^12^^,^^14^^,^^18^^,^^26^^,^^28^^,^^30^^,^^31^^,^^34^^,^^35^
6. Involvement of patients from the onset of the trial^1^^,^^6^^,^^12^, ^13^, ^14^, ^15^^,^^18^^,^^19^^,^^23^^,^^27^^,^^28^^,^^35^
7. Continuous involvement throughout all stages of the trial^1^^,^^2^^,^^6^^,^^12^^,^^18^^,^^23^^,^^28^^,^^36^^,^^37^
8. Regular touchpoints in the project, e.g. meetings or newsletters^1^^,^^2^^,^^15^^,^^17^^,^^23^^,^^28^
9. Involvement of patient groups and organisations^1^^,^^5^^,^^12^^,^^15^^,^^19^^,^^20^^,^^24^^,^^25^^,^^30^
10. Evaluation of the impact and experience of patient involvement by both patients and researchers^2^^,^^5^^,^^7^^,^^15^^,^^20^^,^^28^^,^^34^

## Discussion

Elements that contribute to better patient involvement practices are quite common for all trial stages. Even though patient involvement strategies and activities taking place at each trial stage differ widely, receptive attitudes from all parties, open communication, and a collaborative setting of involvement are all considered to be elements of success and are generally applicable throughout the whole lifecycle of clinical trials.

In the process of involving patients and caregivers, the selection of patient partners appears to be an essential step. It is highly encouraged to gain a deeper insight into their motivations, both personal and social,^11^ as well as their ethnic and educational backgrounds. This understanding will aid in enhancing the quality of cancer care for future patients.^2^^,^^25^

While it is important to ensure inclusivity of diverse perspectives, a special effort is required to involve patient advocates from minorities,^4^^,^^35^^,^^38^ underrepresented populations, or socioeconomically disadvantaged patients.^7^^,^^15^^,^^17^ For this reason, there is a need for improvement of socioeconomic, racial, or ethnic diversity among patient advocates.^7^ Regardless of their education and background, their voices should be considered strong and essential for the design of cancer studies targeting diverse patient populations.^17^ However, patient representatives who have scientific backgrounds, not necessarily in medical sciences, could feel more confident and more easily adapt to the language and processes of medical research.^25^

Efforts made to formalise and conceptualise patient involvement have led to the set-up of international initiatives such as INVOLVE UK, Patient-Centred Outcomes Research Institute in the United States (PCORI), Canada’s Strategy for Patient-Oriented Research (SPOR),^10^^,^^29^ as well as frameworks such as GRIPP or Guidance for Reporting Involvement of Patients and the Public followed by GRIPP2—reporting checklists,^17^^,^^22^^,^^36^ Mullins’s framework (also known as the 10-step patient engagement framework),^37^ Public Involvement Impact Assessment Framework (PiiAF),^25^ Excelerator framework,^28^ and many others. These tools aim to guide researchers when involving patients and provide sets of methods and principles that help to enhance the transparency and consistency of patient involvement activities, as well as promote good quality reporting. Even though evaluation of the feasibility of such toolkits was not the objective of our literature review, we believe that a more nuanced approach is needed to evaluate their potential. As much as a formal, well-structured approach could be preferred by researchers, patients may benefit from a possibility of informal interactions.^25^^,^^39^

Regardless of the strategy an organisation chooses when involving patients in its cancer research activities, ongoing maintenance and nurturing of patient partner relationships is required, as it is an evolutionary process that calls for a certain degree of flexibility.^4^ As Tivey et al. mentioned, any effort of involving patients and caregivers should be context-dependent.^18^ This suggests the need, first of all, to understand that elements that make patient involvement successful and meaningful in one study might vary from those in another one. Secondly, there is a need for balance between identifying areas of collaboration with patients and setting up a plan of their involvement, and yet leaving space for patients’ feedback on the planning process^23^ and unpredictable outcomes to which such involvement can lead.

### Understanding of the notions of ‘successful’ and ‘meaningful’

Today’s literature illustrates strong examples of successful and meaningful patient involvement experience in clinical studies but mainly defined from the perspective of those who conduct research and not from patients’ perspectives.^15^^,^^23^^,^^29^^,^^37^ The reason might be in the expectations set upon researchers: they are expected to set clear objectives and meet them, and ask relevant research questions and answer them. Therefore, their prerequisites for success are measured by well-established procedures they need to follow in order to achieve concrete deliverables of their work.

For patients, on the other hand, successful and meaningful involvement might take a different form. Their understanding of success is likely to be derived from their personal experiences. It is therefore important to take into account the perspectives and experiences of both patient partners and researchers.^2^^,^^34^^,^^38^

## Limitations

This study has a potential limitation. To better understand the perception of success and meaningfulness in the context of patient involvement in oncology, we created a third search string that helped us identify 8 eligible articles out of a total of 40. However, this research question could also be answered based on other included articles as they contained plenty of examples of meaningful and successful patient involvement practices without necessarily using these two adjectives in the title or abstract.

## Conclusions

Our findings demonstrate that patients and caregivers are most involved in the development stage of clinical trials in oncology. There is, however, a lack of evidence of patient involvement in the early and final stages. This may suggest the need for a more systematic and continuous involvement throughout the entire lifecycle of research. Their input, if incorporated from the onset of a clinical trial, can help not only to optimise the study design and improve the quality of patient-facing documents but also to define unmet needs, formulate research priorities of the patient population, and aid the dissemination strategy.

Preparation for patient involvement and the selection of patient partners is found to be an important step of the entire process. In addition to clearly defined roles and expectations, there is a need to assess their motivations before getting involved and to understand their personal experiences. This might not only help to select suitable candidates but also to strengthen the motivation of the research teams and identify the training needs of patients.

Approaches to patient involvement appear to be highly context-dependent. Only by taking into account general principles such as respectful and open communication, transparency, regular project updates, and the creation of a trustworthy, collaborative setting can researchers achieve a successful and meaningful patient involvement experience.

Finally, a wide range of supporting tools and models were reported to help researchers navigate their patient involvement journey. Further research is needed to gain insight into the effectiveness of such tools, the resources required for their implementation, and their cost-effectiveness for study sponsors.
