# Supplementary material for: Elements of successful patient involvement in clinical cancer trials: a review of the literature
Source: ESMO Open. 2024 Mar 15;9(4):102947. doi: 10.1016/j.esmoop.2024.102947 (PMC10959641; doi:10.1016/j.esmoop.2024.102947)
Supplement: Supplementary data [file mmc1.docx]

**Supplementary files**

Table 1. Detailed search strategy for research articles in patient involvement in oncological clinical studies

| **Database Name** | **Search Strategy** | **Number of results** |
| --- | --- | --- |
| **PubMed** | "patient participation"[majr] OR (participation[title] AND "patient involvement"[title] OR "patient engagement"[title])) AND (("Clinical Trials as Topic"[Mesh] AND neoplasms[mesh]) OR ("clinical trial*"[title] AND (cancer*[title] OR neoplas*[title] OR tumor*[title] OR tumour*[title] OR oncolog*[title]))) AND ("2012"[Date - Publication] : "3000"[Date - Publication] | **227** |
| **PubMed** | ("patient participation"[majr] OR (participation[tiab] AND "patient involvement"[tiab] OR "patient engagement"[tiab])) AND (("Clinical Trials as Topic"[Mesh] AND neoplasms[mesh]) OR ("clinical trial*"[tiab] AND (cancer*[tiab] OR neoplas*[tiab] OR tumor*[tiab] OR tumour*[tiab] OR oncolog*[tiab]))) AND ("2012"[Date - Publication] : "3000"[Date - Publication]) AND (Successful*[tiab] OR meaningful*[tiab] OR facilitator*[tiab]) | **40** |
| **PubMed** | Key words: “patient involvement in clinical trials in cancer”  Filters: “published between 2012 and 2023” | **2865** |
|  | Total citations: | **3132** |

Table 2. Overview of studies

| **Trial stage** | **Activities taking place at each stage** | **Number of articles** | **Percentage** |
| --- | --- | --- | --- |
| Pre-development | Idea generation, overall project outline, setting research priorities, PRC approval | 15 | 45% |
| Development | Protocol & PIS/IC design, fundraising, contracts | 28 | 85% |
| Execution | Recruitment, Trial Steering Committee, IDMC | 22 | 67% |
| Dissemination | Publication, communication of study results to participants | 15 | 45% |
| Whole trial lifecycle | All stages: from pre-development to dissemination of trial results | 22 | 67% |

Table 3. Elements of successful patient involvement practice in cancer studies

| **Creating an environment for involving patients** | 1. Clear, explicitly described (documented) expectations, role and responsibilities of team members, incl. patients ^1–10^ 2. Recruitment plan/strategy ^8,9,11^ 3. Advertising on social media networks to find patient partners ^12^ 4. Orientation plans and onboarding documents ^6,7,9^ 5. Communication plan and patient involvement procedure clear to all stakeholders and embedded in the whole research process ^1,2,5,9,13^ 6. Policies that require full disclosure, transparency, accountability ^2^ 7. Terms of References ^7,8,13^ 8. Well-defined strategic objectives of the group/committee/advisory board ^2,13^ 9. Space for a discussion so that patients could participate in the planning process ^7^ 10. Clearly communicated goals of the research and milestones of expected input from patient advisors ^6,14^ 11. Explanation on what data will be shared throughout the project ^2^ 12. Understanding of personal motivations of patients before they get involved, e.g. genuine interest in the topic, altruistic motivation ^1,3,15,16^ 13. Lived experience of cancer ^12^ 14. Involvement of experienced patient partners with health literacy skills and understanding of research environment ^16–18^ 15. Input of multiple patients, incl. under-represented, to ensure diversity ^1,2,5,6,12,13,16,19–22^ 16. Appointment of a patient involvement facilitator/team – (non-clinician) ^8,12,13,19^ 17. Appointment of a scientific mentor or a health care professional “buddy” ^10,14^ 18. Peer support plan – mentoring of other patients, e.g. through an online platform or partnering with a patient organisation ^7^ 19. Building on existing community structures, e.g. community groups ^16^ 20. Inclusion of patient involvement in the budget ^1,3–5,7,8,11,12,16^ 21. Available pool of patient contributors with appropriate skills ^18^ |
| --- | --- |
| **In the process of patient involvement** | 1. Documented decisions in records of proceedings during the process ^4^ 2. Supporting tools and models for researchers (e.g. GRIPP, GRIPP2, CHAN model, PCORI, SPOR, etc.) ^1,3,4,6–11,14,16,20,23–25^ 3. Training and/or supporting information materials for patients ^2–5,8,10,11,14,21–23,27–29^ 4. Communication skills training in the education of patient advocates ^21^ 5. Involvement from the onset of the trial ^2,5,7,8,12–15,21,23,30,31^ 6. Continuous patient involvement in trials throughout all stages of the trial ^1,2,7,8,13,14,23–25^ 7. Option for patients to get involved at different stages of trials ^2^ 8. Patients in committees and trial management groups ^6,13,16,18,25,30,32^ 9. Patient committees and advisory boards ^6,8–10,32^ 10. (Facilitated) focus group discussions ^1,5,8,12,28–31^ 11. Individual assignments besides group discussions ^1^ 12. Options for seriously ill patient partners to rest during group discussions ^7^ 13. Consider emotional well-being at the meetings ^4^ 14. Shared patients’ stories and experiences to strengthened motivation of the research team ^6,8,13^ 15. Regular touchpoints in the project, e.g. meetings or newsletters ^1,6–8,12,13^ 16. Face-to-face meetings for collaborative spirit ^7,8,14^ 17. Face-to-face meetings in combination with a possibility to work on the project materials from home ^1^ 18. Teleconferences and online events to lessen logistical barriers ^12,23,30^ 19. Opportunity to debrief after each meeting and make suggestions that would improve subsequent meetings ^18^ 20. Involvement of patient groups and organisations ^12–14,16,29,30,32,33^ 21. Taking time to explain things, incl. statistical aspects and study findings ^14,16^ 22. Glossary, communication in lay language ^2^ 23. Leave space for informal interactions ^16,18^ 24. Careful moderating when disagreements occurred ^6^ 25. Patient area on the website where patients could comment on the design, layout, and content of patient-facing documents ^13^ 26. Patient contributors knew each other and had worked together previously ^18^ |
| **After patient involvement** | 1. Evaluation of impact and experience of patient involvement ^1,8,10,12,22,26,32^ 2. Communication to patient partners about how their feedback was implemented ^1,32^ 3. Ability to maintain long-term collaborations between researchers and patient partners ^1^ 4. Acknowledgement of patients in publication ^32^ |
| **Applicable to any stage of involving patients** | 1. Reciprocal, trustworthy relationship between patient partners and researchers ^1,2,5^ 2. Open dialogue, clear communication ^2,4,7,10,21,23^ 3. Mutual respect and co-learning ^2,5,6,10,23^ 4. Open and trustworthy atmosphere, collaborative setting ^1,4,8^ 5. Adjusted approach to patient's needs, e.g. in case of bad sight or if not confident with the use of technology ^3,23^ 6. Encouraging graduate students, early career scientists, and junior staff members to engage in patient involvement activities ^8,10,19^ 7. Understanding that patient partners may be personally affected ^4,16^ |

***References***

1. Hovén E, Eriksson L, Månsson D’Souza Å, et al. What makes it work? Exploring experiences of patient research partners and researchers involved in a long-term co-creative research collaboration. *Res Involv Engagem*. 2020;6:33. doi:10.1186/s40900-020-00207-4

2. Michaud S, Needham J, Sundquist S, et al. Patient and Patient Group Engagement in Cancer Clinical Trials: A Stakeholder Charter. *Curr Oncol*. 2021;28(2):1447-1458. doi:10.3390/curroncol28020137

3. Høeg BL, Tjørnhøj-Thomsen T, Skaarup JA, et al. Whose perspective is it anyway? Dilemmas of patient involvement in the development of a randomized clinical trial - a qualitative study. *Acta Oncol*. 2019;58(5):634-641. doi:10.1080/0284186X.2019.1566776

4. Skovlund PC, Nielsen BK, Thaysen HV, et al. The impact of patient involvement in research: a case study of the planning, conduct and dissemination of a clinical, controlled trial. *Res Involv Engagem*. 2020;6:43. doi:10.1186/s40900-020-00214-5

5. Pii KH, Schou LH, Piil K, Jarden M. Current trends in patient and public involvement in cancer research: A systematic review. *Health Expect*. 2019;22(1):3-20. doi:10.1111/hex.12841

6. Smith AB, Lee JR, Lawrence SO, et al. Patient and public involvement in the design and conduct of a large, pragmatic observational trial to investigate recurrent, high-risk non-muscle-invasive bladder cancer. *Cancer*. 2022;128(1):103-111. doi:10.1002/cncr.33897

7. Fox G, Fergusson DA, Foster M, et al. Building a Platform for Meaningful Patient Partnership to Accelerate “Bench-to-Bedside” Translation of Promising New Therapies. *Healthc Q*. 2022;24(SP):74-79. doi:10.12927/hcq.2022.26770

8. Foster M, Fergusson DA, Hawrysh T, et al. Partnering with patients to get better outcomes with chimeric antigen receptor T-cell therapy: towards engagement of patients in early phase trials. *Res Involv Engagem*. 2020;6:61. doi:10.1186/s40900-020-00230-5

9. Needham J, Taylor J, Nomikos D. Integrating Patient-Centred Research in the Canadian Cancer Trials Group. *Curr Oncol*. 2021;28(1):630-639. doi:10.3390/curroncol28010062

10. Ciccarella A, Staley AC, Franco AT. Transforming research: engaging patient advocates at all stages of cancer research. *Ann Transl Med*. 2018;6(9):167. doi:10.21037/atm.2018.04.46

11. Fouad MN, Acemgil A, Bae S, et al. Patient Navigation As a Model to Increase Participation of African Americans in Cancer Clinical Trials. *J Oncol Pract*. 2016;12(6):556-563. doi:10.1200/JOP.2015.008946

12. Nicholas OJ, Joseph O, Keane A, et al. Patient and Public Involvement Refines the Design of ProtOeus: A Proposed Phase II Trial of Proton Beam Therapy in Oesophageal Cancer. *Patient*. 2021;14(5):545-553. doi:10.1007/s40271-020-00487-8

13. Green S, Tuck S, Long J, et al. ReIMAGINE: a prostate cancer research consortium with added value through its patient and public involvement and engagement. *Res Involv Engagem*. 2021;7(1). doi:10.1186/s40900-021-00322-w

14. Beyer K, MacLennan SJ, Moris L, et al. The Key Role of Patient Involvement in the Development of Core Outcome Sets in Prostate Cancer. *Eur Urol Focus*. 2021;7(5):943-946. doi:10.1016/j.euf.2021.09.008

15. Hague C, Foran B, Hall E, et al. Patient Involvement in the Design of a Phase III Trial Comparing Intensity-modulated Proton Therapy and Intensity-modulated Radiotherapy for Oropharyngeal Cancer. *Clin Oncol (R Coll Radiol)*. 2018;30(5):274-276. doi:10.1016/j.clon.2018.01.018

16. South A, Hanley B, Gafos M, et al. Models and impact of patient and public involvement in studies carried out by the Medical Research Council Clinical Trials Unit at University College London: findings from ten case studies. *Trials*. 2016;17:376. doi:10.1186/s13063-016-1488-9

17. Perlis N, Finelli A, Lovas M, et al. Creating patient-centered radiology reports to empower patients undergoing prostate magnetic resonance imaging. *Can Urol Assoc J*. 2021;15(4):108-113. doi:10.5489/cuaj.6585

18. Croudass A, Stephens R. The value of the patient and public contribution to cancer research UK’s review of covid-19 impact on its clinical research portfolio. *Res Involv Engagem*. 2021;7(1):35. doi:10.1186/s40900-021-00279-w

19. Van Hemelrijck M, Peters V, Loong JF, et al. The importance of patient and public involvement in cancer research: time to create a new job profile. *Future Oncol*. 2021;17(28):3667-3670. doi:10.2217/fon-2021-0489

20. Cruz Rivera S, Stephens R, Mercieca-Bebber R, et al. “Give Us The Tools!”: development of knowledge transfer tools to support the involvement of patient partners in the development of clinical trial protocols with patient-reported outcomes (PROs), in accordance with SPIRIT-PRO Extension. *BMJ Open*. 2021;11(6):e046450. doi:10.1136/bmjopen-2020-046450

21. Katz ML, Archer LE, Peppercorn JM, et al. Patient advocates’ role in clinical trials: perspectives from Cancer and Leukemia Group B investigators and advocates. *Cancer*. 2012;118(19):4801-4805. doi:10.1002/cncr.27485

22. Mutebi M, Scroggins D, Simmons V, Oti NO, Hammad N. Engaging Patients for Clinical Trials in Africa: Patient-Centered Approaches. *JCO Glob Oncol*. 2020;6:942-947. doi:10.1200/JGO.19.00190

23. Tivey A, Huddar P, Shotton R, et al. Patient engagement in melanoma research: from bench to bedside. *Future Oncol*. 2021;17(28):3705-3716. doi:10.2217/fon-2020-1165

24. Davies-Teye BB, Medeiros M, Chauhan C, Baquet CR, Mullins CD. Pragmatic patient engagement in designing pragmatic oncology clinical trials. *Future Oncol*. 2021;17(28):3691-3704. doi:10.2217/fon-2021-0556

25. Buckley C, Treweek S, Laidlaw L, Shiely F. Patient and Public Involvement (PPI) in outcome selection in breast cancer and nephrology trials. *Trials*. 2023;24(1):93. doi:10.1186/s13063-022-06980-9

26. Faulkner SD, Somers F, Boudes M, Nafria B, Robinson P. Using Patient Perspectives to Inform Better Clinical Trial Design and Conduct: Current Trends and Future Directions. *Pharmaceut Med*. 2023;37(2):129-138. doi:10.1007/s40290-022-00458-4

27. Sacristán JA, Aguarón A, Avendaño-Solá C, et al. Patient involvement in clinical research: why, when, and how. *Patient Prefer Adherence*. 2016;10:631-640. doi:10.2147/PPA.S104259

28. Fallowfield L, Francis A, Catt S, Mackenzie M, Jenkins V. Time for a low-risk DCIS trial: harnessing public and patient involvement. *Lancet Oncol*. 2012;13(12):1183-1185. doi:10.1016/S1470-2045(12)70503-X

29. Zaharoff B, Cipra S. Improving Oncology Clinical Trial Participation and Experience. *Trends Cancer*. 2018;4(12):793-796. doi:10.1016/j.trecan.2018.10.007

30. Batten LM, Bhattacharya IS, Moretti L, et al. Patient advocate involvement in the design and conduct of breast cancer clinical trials requiring the collection of multiple biopsies. *Res Involv Engagem*. 2018;4:22. doi:10.1186/s40900-018-0108-0

31. Powell JR, Murray L, Burnet NG, et al. Patient Involvement in the Design of a Randomised Trial of Proton Beam Radiotherapy Versus Standard Radiotherapy for Good Prognosis Glioma. *Clin Oncol (R Coll Radiol)*. 2020;32(2):89-92. doi:10.1016/j.clon.2019.09.049

32. Vasu S, Holtan SG, Shimamura A, et al. Bringing Patient and Caregivers Voices to the Clinical Trial Chorus: A Report From the BMT CTN Patient and Caregiver Advocacy Task Force. *Transplant Cell Ther*. 2023;29(1):5-9. doi:10.1016/j.jtct.2022.10.016

33. Rocque GB, Williams CP, Andrews C, et al. Patient perspectives on chemotherapy de-escalation in breast cancer. *Cancer Med*. 2021;10(10):3288-3298. doi:10.1002/cam4.3891
